# Infections in Kidney Transplant Recipients: Perspectives in French Caribbean

**DOI:** 10.3390/microorganisms12122390

**Published:** 2024-11-22

**Authors:** Laurène Tardieu, Gary Doppelt, Muriel Nicolas, Violaine Emal, Pascal Blanchet, Samuel Markowicz, Valérie Galantine, Pierre-Marie Roger, Joëlle Claudéon, Loïc Epelboin

**Affiliations:** 1 Service de Maladies Infectieuses et Tropicales, Centre Hospitalier Universitaire, 97139 Les Abymes, Guadeloupe, France; samuel.markowicz@chu-guadeloupe.fr (S.M.); pierre-marie.roger@chu-guadeloupe.fr (P.-M.R.); 2 Service de Radiologie, Centre Hospitalier de Cayenne, 97306 Cayenne, Guyane Française, France; 3 Service de Microbiologie, Centre Hospitalier Universitaire, 97139 Les Abymes, Guadeloupe, France; muriel.nicolas@chu-guadeloupe.fr; 4 Service de Néphrologie, Centre Hospitalier Universitaire, 97261 Fort-de-France, Martinique, France; violaine.emal@gmail.com; 5 Service d’Urologie, Centre Hospitalier Universitaire, 97139 Les Abymes, Guadeloupe, France; pascal.blanchet@chu-guadeloupe.fr; 6 Service de Néphrologie, Centre Hospitalier Universitaire, 97139 Les Abymes, Guadeloupe, France; valerie.aloph@chu-guadeloupe.fr (V.G.); joelle.claudeon@chu-guadeloupe.fr (J.C.); 7 Service des Maladies Infectieuses et Tropicales, Centre Hospitalier de Cayenne, 97300 Cayenne, Guyane Française, France; epelboincrh@hotmail.fr; 8 CIC Inserm 1424, Centre Hospitalier de Cayenne, 97300 Cayenne, Guyane Française, France

**Keywords:** transplant infectious diseases, kidney transplantation, nocardiosis, phaeohyphomycosis

## Abstract

Few studies have focused on the infectious complications in kidney transplant recipients in tropical regions, particularly in the Caribbean. The primary objective of this study was to determine the incidence of bacterial, fungal, and parasitic infections in kidney transplant recipients in the French Caribbean and French Guiana. We included all patients who received a kidney transplant at the University Hospital of Guadeloupe between January 2014 and October 2016, with post-transplant follow-up in the French Caribbean. A total of 91 patients were included, of whom 57 developed an infectious event during follow-up. When infections were documented (94/111), bacterial infections were the most frequent (79/94), followed by fungal (11/94) and parasitic infections (4/94). Four cases of nocardiosis were identified (4/79). Phaeohyphomycosis was the most common fungal infection (7/11). In a multivariate analysis, the female gender and diabetes mellitus at the time of transplant were significantly associated with a higher risk of infection. This study is the first to describe the epidemiology of infections in kidney transplant recipients in the Caribbean and to analyze the potential risk factors. We reported a similar profile of bacterial infections to that which were observed in the European and American studies. However, we found a higher incidence of tropical infections, such as nocardiosis and phaeohyphomycosis, which highlights the need for heightened awareness among healthcare teams to ensure earlier and more appropriate treatment. Further studies focusing on these rare tropical infections are necessary to better understand their risk factors

## 1. Introduction

Approximately 70% of kidney transplant recipients (KTRs) experience infectious events within the first three years after transplantation, particularly within the initial 24 months [1]. Infection is the second leading cause of death among recipients with functioning allografts, with cardiovascular disease being the primary cause [2]. Advances in immunosuppressive therapies, anti-infective prophylaxis, and patient characteristics have transformed the epidemiology of infections in recent decades, resulting in an older and more comorbid transplant population [3]. These insights are essential for optimizing treatment, including reducing diagnostic delays, adjusting anti-rejection therapy, and initiating appropriate anti-infective interventions.

Despite progress, 40 to 70% of patients still experience at least one infection during the first two years of follow-up [1,3,4]. Urinary tract infections (UTIs) are the most common, followed by respiratory tract infections (RTIs), skin and soft tissue infections, and gastrointestinal infections [2,5]. Well-established risk factors include age, female sex, dialysis duration, and the occurrence of rejection [4,5].

Survival rates and graft function have significantly improved in KTRs over the past few decades, primarily due to the better management of rejection risks and a more comprehensive understanding of infection risks [3,4]. However, infections remain the leading cause of morbidity and mortality among KTRs, particularly in middle-income countries like Brazil [6]. The lack of transplant registries complicates the systematic study of post-transplant infections in tropical countries. Currently, only one center is authorized to perform transplants in the French Caribbean. In the Dutch Antilles, a transatlantic program was initiated in 1998 to facilitate the placement of end-stage renal disease patients on the Eurotransplant waiting list, as no transplant centers exist in these areas [7]. Despite the growing regional transplant activity, many KTRs living in the outermost regions and overseas territories undergo transplantation in mainland Europe, with follow-up care shared between their transplant center and healthcare professionals in their home region.

Most of the available data come from individual physicians in a limited number of centers. It is estimated that infections complicate the course of 50–75% of transplant recipients in tropical countries, with mortality rates ranging from 20 to 60% [8,9]. Contributing factors include poor hygiene, delayed presentations, limited knowledge of the range of organisms present in these regions, and restricted access to life-saving antimicrobial agents [9].

No study has specifically investigated the infectious complications in KTRs in the French Caribbean, which includes Martinique, Guadeloupe, Saint Martin, and Saint Barthelemy. Existing studies in this region have focused mainly on transplant activity without providing detailed information on infections [10,11]. Notably, the University Hospital of Guadeloupe is the only transplant center serving the French Caribbean and French Guiana. Understanding the local epidemiology of infections is crucial to reducing diagnostic delays and optimizing therapeutic management.

The primary objective of this study was to investigate the epidemiology of bacterial, fungal, and parasitic infections among KTRs in the French Caribbean. The secondary objectives included analyzing the risk factors associated with infections in this population and describing the associated mortality.

## 2. Materials and Methods

### 2.1. Settings

The study was conducted in the French overseas territories of Martinique, Guadeloupe, Saint Barthelemy, Saint Martin, and French Guiana. These territories, located in the Americas, experience an intertropical climate with alternating wet and dry seasons. Martinique, Guadeloupe, Saint Barthelemy, and Saint Martin are situated in the Lesser Caribbean, while French Guiana is on the northeastern coast of South America, bordered by Brazil and Suriname. Kidney transplant patients in these regions typically undergo transplantation either at the University Hospital of Guadeloupe or mainland France, as there are no transplant departments in Martinique, Saint Barthelemy, Saint Martin, or French Guiana. All patients are registered in the French national transplant registry, with those in the Caribbean being affiliated with the Guadeloupe center.

Due to logistical challenges, most kidneys are procured in Guadeloupe to minimize cold ischemia time. In rare instances, kidneys may be exchanged between mainland France and the French West Indies based on immunological compatibility. Some patients opt to register in mainland France, but this requires urgent travel upon receiving a transplant offer, which complicates logistics. Waiting times for transplants in these regions are comparable to those in other French transplant centers.

### 2.2. Transplant Procedure

Kidneys harvested are placed on a perfusion machine, similar to the practice in mainland France. Ureterovesical anastomosis is then performed using the Lich Grégoire technique, with double-J stents systematically inserted intraoperatively and removed one month after transplantation in the absence of complications. Tacrolimus blood levels were monitored on postoperative day 7 and monthly thereafter. During follow-up, patients were seen weekly for the first month, biweekly during the second month, monthly until the sixth month, and every three months thereafter.

All transplant patients received antibiotic prophylaxis with amoxicillin/clavulanic acid (Augmentin 1 g) administered just before the transplantation, three hours after unclamping, and then 1g twice daily until urinary catheter removal. Valganciclovir was administered to all patients with positive CMV serology (donor or recipient), starting on day 5 and adjusted according to renal function, for 90 to 180 days. Patients with negative CMV serology and a negative donor did not receive Valganciclovir. Sulfamethoxazole/trimethoprim prophylaxis was provided from day 1, continuing for at least 90 days. The dose was adjusted according to the renal function, typically 80/400 mg daily.

Pre-transplant preventive measures align with those recommended in mainland France. Vaccination for yellow fever is recommended (but not obligatory) for patients planning to live or travel to French Guiana.

### 2.3. Study Design and Population

This multicenter retrospective cohort study included patients from the University Hospital of Guadeloupe and Martinique. All patients who received a kidney transplant between 1 January 2014 and 31 October 2016 were included. Patients residing in French Guiana, Saint Martin, or Saint Barthelemy were followed up in Guadeloupe, while patients from Martinique were followed in Martinique.

Patients were excluded if they were pediatric (<18 years), residing outside the French Caribbean or French Guiana, or lost to follow-up (defined as absence of post-transplant follow-up within 30 days due to relocation or non-compliance). Combined transplants were also excluded. Data collection covered a 24-month post-transplant period, corresponding to the timeframe with the highest incidence of complications [3].

### 2.4. Data Collection

Demographic, clinical, and biological data were collected retrospectively from electronic and paper records at the University Hospitals of Guadeloupe and Martinique. Collected data included pre-transplant characteristics (age, sex, BMI, comorbidities such as hypertension, diabetes, obesity, number of transplants, nephropathy, dialysis duration); transplant-related variables (cold ischemia time, donor type, immunological risk, treatment); and post-transplant outcomes (rejection, infections, neutropenia, lymphopenia, survival). In cases of death, the cause was determined from the medical records.

All bacterial, fungal, and parasitic infections were documented, regardless of hospitalization status. However, the incidence of viral infections was not recorded due to incomplete virological data. CMV serostatus was documented at the time of transplantation for all patients, and these data have been summarized in the patient characteristics table. Routine screening for other viral infections was not performed. Data on CMV replication and BK viremia were collected during infection episodes, when available.

A positive bacterial or fungal culture in the graft preservation fluid alone was not considered sufficient to classify a case as an infection. Only cases in which recipients displayed clinical symptoms or had additional corroborating positive cultures (e.g., blood, urine) were classified as infections.

### 2.5. Ethical Considerations

The study adhered to reference methodology No. 4 (MR-004) under the European General Data Protection Regulation and was approved by the Ethics Commission of the University Hospital of Guadeloupe on 13 September 2021 (Registration number: A65_21_09_13_TREIN).

### 2.6. Definitions

Infectious events were defined as any suspected or confirmed infection requiring treatment, whether or not hospitalization was necessary. Parasitic infections were included if immunosuppressive therapy was reduced, even in the absence of specific treatment.

- Cases: Patients with at least one infectious event;
- Controls: Patients with no infectious event;
- **UTIs**: Cystitis was defined as a bacterial count > 10^5^ CFU/mL, with dysuria, frequency, or urgency, while pyelonephritis required a bacterial count > 10^5^ CFU/mL, with fever and specific clinical signs (lumbar pain, graft pain, etc.) [12];
- **Lymphopenia:** Lymphocyte count < 1500/mm^3^, following laboratory standards;
- **Neutropenia:** Neutrophil count < 1500/mm^3^, following laboratory standards;
- **BK viremia:** Defined as a BK virus DNA load of ≥10^3^ copies/mL (3 Log10 copies/mL) in the blood detected through quantitative PCR;
- **High immunological risk**: Defined by donor-specific anti-HLA antibodies > 2000 MFI;
- **Phaeohyphomycosis**: Diagnosed by culture or histopathological evidence of dematiaceous fungi [13];
- **Lymphocele infection**: Fluid collection in the transplant space, diagnosed by pathogen-positive puncture [14];
- Rejection: Confirmed by pathological results from an allograft biopsy.

### 2.7. Data Analysis

Statistical analyses were performed using Microsoft Excel (v.16.60), GraphPad (v. 9.3.1), and R 4.1.1. Qualitative variables were described as proportions, and continuous variables were presented as means ± standard deviations or medians with interquartile ranges, depending on normality. Bivariate analyses were conducted using χ^2^ tests for qualitative variables, Student’s *t*-test for normally distributed quantitative variables, and the Mann–Whitney U test for non-normally distributed variables. A *p*-value < 0.05 was considered statistically significant. Logistic regression was used to identify risk factors for infection, with variables showing *p* ≤ 0.2 in the bivariate analysis included in the multivariate model. Sensitivity analyses were also performed by including clinically relevant variables that were not statistically significant to assess the robustness of the multivariate model.

## 3. Results

### 3.1. Global Patient Characteristics

**Figure 1:**
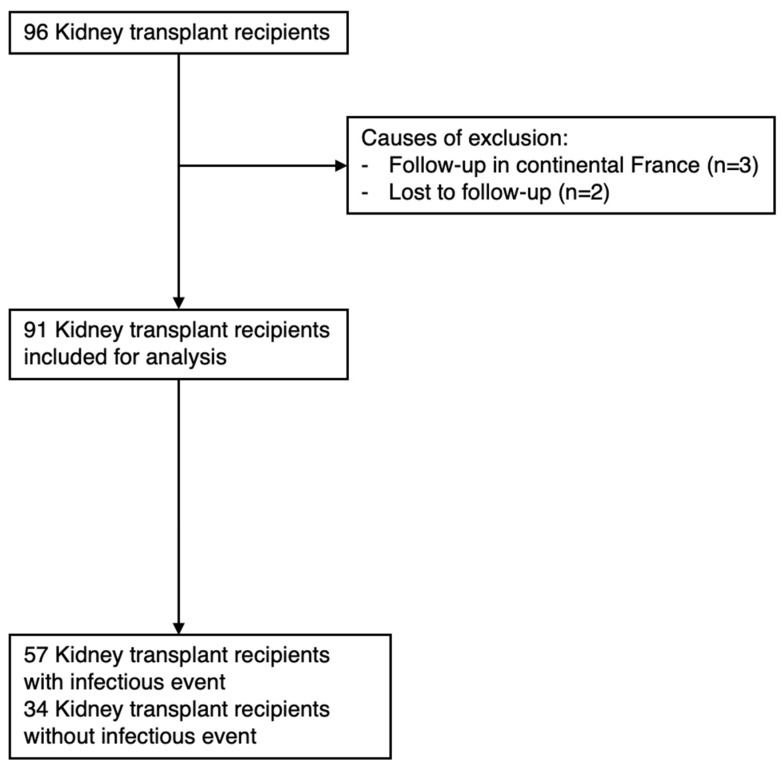
Flowchart.

Between 1 January 2014 and 31 October 2016, 96 patients were transplanted consecutively at the University Hospital of Guadeloupe. Two patients were excluded due to missing data, and three were excluded after relocating to mainland France during follow-up (see flowchart in Figure 1). As a result, 91 kidney transplant recipients (KTRs) were included in the study. The clinical characteristics of the 91 KTRs are summarized in Table 1. The male-to-female sex ratio was 2:1, and the median age at inclusion was 52 years (IQR [47–59]). Cardiovascular comorbidities were present in 21% (19/89) of patients, specifically diabetes mellitus. Most KTRs lived in Guadeloupe (78%, 71/91), followed by Martinique (14%, 13/91), French Guiana (6%, 5/91), and Saint Martin (2%, 2/91). All patients received their first kidney transplant, with 94% of donors being deceased and the rest living. The median hospitalization duration post-transplant was 22 days (IQR [14–28.5]). Patients were considered to be at high immunological risk in 59% (54/91) of cases. During follow-up, 20% (18/89) of KTRs experienced transplant rejection. All patients received induction treatment with anti-thymocyte globulin (ATG), with 61% (55/90) of patients subsequently receiving tacrolimus, mycophenolate mofetil, and corticosteroids. The remaining patients were treated with ciclosporin and mycophenolate mofetil post-induction.

### 3.2. Infected Patient Characteristics

#### 3.2.1. Demographics

Among the 91 patients, 57 presented with at least one infectious event during follow-up. There were significantly more women in the group of infected patients (44% vs. 33%, *p* = 0.005) (Table 1). There was no significant difference in age between the infected and uninfected groups (*p* = 0.899). The median BMI was 25 (IQR [23–28]), with no significant difference between groups.

#### 3.2.2. Comorbidities

Diabetes mellitus at the time of transplantation was present in 21% of patients and was significantly more frequent among those with infections (*p* = 0.007). The median cold ischemia time was 20 h, with no significant difference between the two groups (*p* = 0.627).

### 3.3. Description of Infectious Events

#### 3.3.1. Timing and Sites of Infections

**Figure 2:**
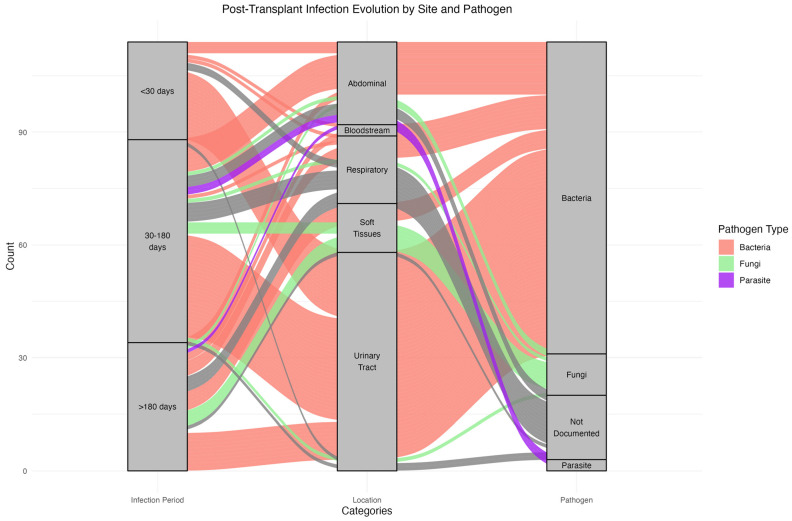
Alluvial Plot Distribution of Infection Types and Pathogens Over Time in Kidney Transplant Recipients.

The median time from transplantation to the first documented infection was 34 days (IQR [13–120]) (Table 2). Infections were most frequent within the first 30 days post-transplant (45.5%), predominantly bacterial and mainly involving the urinary tract. Between 30 days and 180 days post-transplant, infections accounted for 35.5% of cases, with a notable presence of fungal pathogens in respiratory and soft tissue sites. Infections after 180 days were less common (19%) and included bacterial, fungal, and parasites, as well as a more balanced distribution of pathogens. These observations are shown in Figure 2, detailing infection timing, site, and pathogen type.

#### 3.3.2. Recurrent Infection Patterns and Pathogen Consistency

Among patients with multiple infection episodes, the patterns of infection sites and pathogen consistency are summarized in Table 3. Urinary tract infections were the most frequently recurring site, with similar pathogens identified in 50% of cases across episodes. Pulmonary infections, though less frequent, did not show pathogen similarity across episodes. In cases involving infections at multiple sites (such as digestive and lymphocele infections), pathogen diversity was higher, particularly in patients with four or more episodes, where different pathogens were identified in up to 73% of cases. *Klebsiella pneumoniae* producing extended-spectrum beta-lactamase (ESBL-KP) was the most frequently identified recurrent pathogen.

### 3.4. Microbiological Characteristics

**Figure 3:**
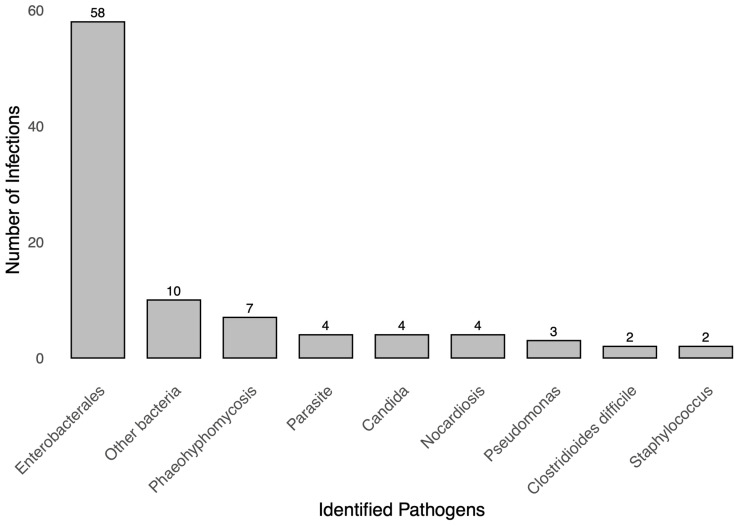
Microbiological Distribution of Infections in Kidney Transplant Recipients.

Microbiological data of the infections are summarized in Table 4 and illustrated in Figure 3. Of the 115 infections, 82% were microbiologically documented. Respiratory infections were the least documented, with only 59% confirmed microbiologically. Bacterial infections accounted for 84% of cases, fungal infections for 12%, and parasitic infections for 4%. Among the bacterial infections, *Klebsiella pneumoniae* producing extended-spectrum β-lactamase (ESBL) was the most common pathogen (28.8%), followed by *Escherichia coli* (22.5%) and non-ESBL *Klebsiella pneumoniae* (7.5%). Among the bacterial isolates, *Staphylococcus aureus* was identified, and none of these isolates were resistant to oxacillin, indicating the absence of MRSA in our cohort. Four cases of *Nocardia* infections were identified, including one case of disseminated disease. Fungal infections were predominantly due to phaeohyphomycosis (63.6%) involving soft tissues. Digestive infections were mostly caused by *Cryptosporidium* (*n* = 3), followed by *Clostridioides difficile* (*n* = 2).

### 3.5. Infection Risk Factors

In bivariate analysis, the female sex (*p* = 0.005) and diabetes mellitus at the time of transplantation (*p* = 0.007) were significantly associated with a higher risk of developing post-transplant infections. There was no association between infection risk and induction or maintenance immunosuppressive regimens, acute rejection episodes, or dialysis duration before transplantation. In multivariate analysis, independent factors associated with infection included the female sex (OR 4.33, 95% CI [1.40–13.37]) and diabetes mellitus at the time of the transplant (OR 7.65, 95% CI [1.59–36.81]) (Table 5).

A sensitivity analysis was conducted by including two clinically relevant but statistically non-significant variables, namely hypertension and length of hospital stay. The results showed that neither variable reached statistical significance (hypertension: OR = 0.31, *p* = 0.31; length of hospital stay: OR = 1.04, *p* = 0.15), indicating that these factors did not significantly impact infection risk in our study population.

### 3.6. Outcomes

During the 24-month follow-up period, 12% (11/91) of patients died, with no significant difference in mortality between the infected and uninfected patients (*p* = 0.5). Among those who experienced an infectious event, 14% (8/57) died during follow-up, with 62.5% of deaths attributable to infection. The most common cause of death was pneumonia (*n* = 3), with pathogens identified as *Enterobacter cloacae*, *Pseudomonas aeruginosa*, and *Nocardia farcinia*.

## 4. Discussion

### 4.1. Clinical Features

We presented the results of a multicenter cohort analysis conducted on 91 kidney transplant recipients (KTRs). This is the first study describing infections in kidney transplant patients in the French Caribbean and one of the few studies conducted in tropical regions [6,9,15,16,17]. In this study, 63% of patients developed an infection during follow-up, a rate similar to the 45–70% reported in the literature [3,4,5]. Considering that viral infections were not included, our incidence rate may be underestimated. The average time to onset of infections is also comparable with existing data, occurring predominantly in the first year post-transplant, between 6 and 12 months [3].

The predominance of female patients in the infected group is consistent with previous findings, as the female sex is a known risk factor for infection, particularly urinary tract infections (UTIs) [5]. Established risk factors for infections in KTRs include age, the female sex, time on dialysis, diabetes, and the occurrence of rejection [5]. In our cohort, we found significantly more infections among women and diabetic patients. Other known risk factors may not have emerged as significant due to the relatively small sample size. Although immunosuppressive induction with ATG is a known risk factor for infection, all patients in our cohort received ATG, which prevented us from assessing its impact on infection risk [18].

### 4.2. Types of Infections

UTIs were the most common infections, accounting for 53% of all cases. The incidence of UTIs after kidney transplantation varies from 31% to 85% across studies [12,16], likely reflecting the differences in case definitions and follow-up periods. However, UTIs are consistently the most frequent infection reported.

The high incidence of lymphocele infections may be explained by surgical techniques, particularly the use of uretero–vesical or uretero–ureteral anastomosis, which increases the risk of lymphocele formation. Moreover, transplant teams may not systematically screen for lymphocele infections. Some teams prefer not to perform lymphocele punctures or marsupialization, leading to fewer documented infections. Further studies on this subject would be valuable.

The microbiological spectrum we observed aligns with existing data, with a predominance of Enterobacteriaceae, although *Klebsiella pneumoniae* ESBL was the most common uropathogen rather than *Escherichia coli* [12,19,20,21,22,23].

Up to 24% of infections were caused by ESBL-producing Enterobacteriaceae, a rate comparable to the studies that reported rates ranging from 11% to 53% depending on the region [21,24]. The emergence of multi-drug-resistant Gram-negative bacteria is a global concern, particularly for KTRs, where the delayed initiation of the appropriate antibiotic therapy is associated with high mortality in cases of sepsis [25,26]. Knowledge of local antibiotic resistance patterns is essential for timely and adequate management. Further research into the local risk factors for ESBL infections would be valuable, especially considering the higher prevalence of diabetes in our cohort compared to the American or European populations [27].

A recent study on KTR infections in French Guiana found similar results to ours, with a predominance of UTIs and bacterial infections. However, they also reported endemic fungal infections, with histoplasmosis being the most frequent fungal pathogen [9].

### 4.3. Atypical Incidence of Nocardiosis and Phaeohyphomycosis

Our study reported a 4.3% incidence of nocardiosis, higher than the 0.04% to 1% incidence typically reported in the literature [28,29,30]. Known risk factors for nocardiosis include advanced age, high concentrations of calcineurin inhibitors (CNI), the use of tacrolimus, CMV disease, and prolonged post-transplant hospitalization [31]. The tropical climate, which favors the growth of *Nocardia* spp. in humid environments, may also explain the higher incidence in the French Caribbean [28,32]. Additional studies are needed to explore specific risk factors for nocardiosis in this region.

Similarly, the incidence of phaeohyphomycosis in our cohort (7.6%) was higher than the 0.7% incidence reported in solid organ transplant recipients in the USA over 22 years [33]. The increased incidence could be attributed to the tropical environment and the higher prevalence of diabetes in our population. Phaeohyphomycosis-causing molds are ubiquitous environmental fungi that can cause a range of infections, including skin and subcutaneous lesions, pneumonia, and disseminated disease [34]. Due to the rarity of these infections, further prospective studies are needed to investigate the specific risk factors.

### 4.4. Mortality

The mortality rate in our study (10%) appears higher than in other studies. This may be due to the fragility of our population, which has more limited access to care and, as a result, may experience delayed treatment. Additionally, our patients may have more comorbidities. Further studies are required to examine these factors in more detail.

### 4.5. Limitations

This study has several limitations. First, the retrospective design led to some missing data in the medical records. Second, the relatively small cohort size limits the generalizability of our findings, and it is possible that some infections were missed, particularly those managed in outpatient settings. Microbiological documentation, particularly for lung infections, was also incomplete, a common challenge even among non-KTR patients.

We did not collect virological data, which would have provided a more comprehensive analysis of the infections in KTRs in the French Caribbean. CMV PCR results were only recorded for patients with infections, limiting our ability to analyze CMV infections in the overall cohort.

The follow-up period of 24 months may have been too short to capture later-occurring infections. A longer follow-up would have allowed us to identify infections occurring beyond this period. Additionally, more detailed analyses of kidney function over time would have been beneficial, as impaired kidney function could be a potential risk factor for infections.

## 5. Conclusions

This study is the first to comprehensively analyze bacterial, fungal, and parasitic infections in kidney transplant recipients (KTRs) living in the Caribbean and one of the few conducted in a tropical region. The increasing number of transplants performed in tropical areas, coupled with a growing population of transplant patients traveling to these regions, suggests a likely rise in such infections in the coming years.

Our study highlights that the incidence of common infections in KTRs in the French Caribbean appears to be at least comparable to the existing literature. We have identified similar infection risk factors, such as the female gender and diabetes at the time of transplantation. Furthermore, given the high prevalence of ESBL infections, along with the increased frequency of nocardiosis and phaeohyphomycosis, our findings can equip healthcare practitioners caring for kidney transplant patients with valuable insights into the spectrum of infections they may encounter during follow-up. Future research in these regions should aim to identify the specific risk factors unique to this population.

This study also emphasizes the significance of microbiological sampling for suspicious skin lesions, as it can facilitate the diagnosis of these infections.

## Figures and Tables

**Table 1:** Characteristics of patients.

Clinical Features	N	Missing Data	Total (*n* = 91)	Case (*n* = 57)	Control (*n* = 34)	*p*-Value
Age (years), median [IQR]	91	0	52 [47–59]	52 [47–61]	52 [49–57]	0.899
Sex, No. (%)	91	0				
Male			61 (67.0)	32 (56.1)	29 (85.3)	0.005
Female			30 (33.0)	25 (43.9)	5 (14.7)	
BMI (kg/m^2^), median [IQR]	91	0	25.2 [23–28]	24.9 [22.5–29.3]	25.5 [23–28]	0.487
Diabetes at time of transplant, No. (%)	89	2	19 (21.3)	17 (30.9)	2 (5.9)	0.007
Dialysis time to transplant (months), median [IQR].	88	3	46 [28–82]	45 [22–78]	52 [31–94]	0.488
Territory of residence. No. (%)	91	0				
Guadeloupe			71 (78.0)	46 (80.7)	25 (73.5)	
Martinique			13 (14.3)	7 (12.3)	6 (17.6)	
French Guiana			5 (5.5)	3 (5.3)	2 (5.9)	
Saint Martin			2 (2.2)	1 (1.8)	1 (2.9)	
HBP, No. (%)	89	2	81 (91.0)	48 (87.3)	33 (97.1)	0.150
CMV Positive Serostatus, No. (%)	91	0	74 (81.3%)	48 (84.2%)	26 (76.5%)	0.411
Number of previous transplants, No. (%)	91	0				1
1				1 (1.1)	1 (2.9)	
>1				0 (0.0)	0 (0.0)	
Donor type, No. (%)						
Death	89	2	85 (94.4)	53 (94.6)	32 (94.1)	
Living	89	2	5 (5.6)	3 (5.4)	2 (5.9)	
Cold ischemia time (hour), median [IQR]	84	7	20.0 [17–24]	20.4 [17.5–24.5]	19.3 [14–23.8]	0.627
Positive graft preservation fluid with bacteria or fungus	91	0	22 (24.2)	16 (28.1)	6 (17.6)	0.318
Length of stay after transplantation (days), median [IQR]	90	1	19 [14–28]	19.5 [14–30]	16 [12–24]	0.024
Immunologic risk at transplantation, No. (%)	91	0				0.510
HRI			54 (59.3)	36 (63.2)	18 (52.9)	
FRI			37 (40.7)	21 (36.8)	16 (47.1)	
Acute or chronic rejection during follow-up, No. (%)	89	2	18 (20.2)	12 (21.4)	6 (18.1)	1
Deaths during follow-up	91	0	11 (12.1)	8 (14.0)	3 (8.8)	0.5
Therapeutic characteristics						
Immunosuppressive therapy per transplant, No. (%)	90	1				
ATG Induction			90 (100.0)	57 (100.0)	33 (100.0)	
Ciclosporin—Cellcept			35 (38.9)	20 (35.1)	15 (45.5)	0.37
Tacrolimus—Cellcept—Corticoids			55 (61.1)	37 (67.3)	18 (32.7)	

Note. ATG: anti-thymocyte globulin BMI: body mass index; FRI: low immunological risk; HRI: high immunological risk; HBP: High blood pressure; IQR: interquartile range; M: months; SD: standard deviation.

**Table 2:** Clinical and biological characteristics of infections of 57 patients.

	N	Total
Clinical Features		
Age at infection (years), mean (SD)	57	53.0 (9.8)
Total number of infections, No. average per patient (SD)	116	2.0 (1.5)
Number of patient with 1 infection, No. %		32 (56)
Number of patient with 2 infection, No. %		10 (17)
Number of patient with 3 or more infection, No. %		15 (26)
Median time between transplantation and the first infection, days, median [IQR]	57	34 [13–120]
Time from graft to first documented infection, number of cases per period, number of subjects (%)		
D0–M1		23 (20.7)
Bacterial		23
Fungal		0
Parasitic		0
M1–M6		54 (48.6)
Bacterial		37
Fungal		6
Parasitic		2
M6–M24		34(30.7)
Bacterial		23
Fungal		5
Parasitic		1
Site of infection	57	
Urine		58 (53.2)
Lung		17 (15.6)
Soft tissues		10 (9.2)
Abdominal		11 (10.1)
Lymphocele		11 (10.1)
Blood		1 (0.9)
NA		1 (0.9)
Multiple sites of infections (>1)		
2 sites		24 (42%)
3 sites		16 (28%)
4 sites or more		12 (21%)
Biological features		
Lymphopenia during infection	115	101 (87.8)
Neutropenia during infection	109	9 (8.3)
Microbiological identification, No. (%)	94	
Bacterial		79 (84.0)
Fungal		11 (11.7)
Parasite		4 (4.2)
Concurrent CMV replication, No. (%)	102	10 (9.8)
Concurrent BK viremia replication, No. (%)	94	7 (7.4)
Death		
Deaths during follow-up, No. (%)		8 (14)
Infectious cause		5
Other cause		

Note. BK: BK virus; CMV: cytomegalovirus; IQR: interquartile range; SD: standard deviation; No.: number; %: percentage; NA: not applicable; D0–M1 represents infections occurring in the first month post-transplant; M1–M6 represents months 1 to 6; M6–M24 represents months 6 to 24.

**Table 3:** Summary of Recurrent Infection Patterns, Site Consistency, and Main Pathogens in Patients with Multiple Infection Episodes.

Number of Infections	Type of Recurrent Infection	Number of Patients	Similar Pathogens (%)	Main Pathogen
2	Urinary (only repeated site)	8	4 (50)	ESBL-KP
2	Pulmonary (only repeated site)	4	0 (0)	-
3	Urinary (only repeated site)	4	1 (25)	ESBL-KP
4	Multi-site Infections (with no urinary infection)	1	0 (0)	x
4 or more	Urinary + other sites (digestive, lymphocele, etc.)	11	3 (27)	ESBL-KP

Note: ESBL-KP: Extended-Spectrum Beta-Lactamase producing *Klebsiella pneumoniae.*

**Table 4:** Microbiological characteristics of infections.

			UTI *	Pulmonary	Digestive	Soft Tissues	Lymphocele	Blood	Disseminated
**Total infections**	109	(%)							
**Bacteria**	79	(72)							
Wild type-*E. coli*	17		17						
Others *E. coli*	1		1						
ESBL-KP	23		18	1			4		
Wild type-KP	6		4		1		1		
*Pseudomonas aeruginosa*	3		2	1					
*Enterobacter cloacae*	3		2				1		
*Morganella morganii*	3		1			1	1		
*Haemophilus influenzae*	1			1					
*Enterococcus fecalis*	2		2						
*Salmonella* spp.	1				1				
*Proteus mirabilis*	2		2						
*Providentia* sp.	1		1						
*Clostridioides difficile*	2				2				
*Acinetobacter baumanii*	3		2					1	
*Citrobacter freundii*	1						1		
*Stenotrophomonas maltophilia*	1		1						
*MSSA*	2					1	1		
*Nocardia* sp.	4			2		1			1
*Chryseobacterium*	1		1						
*Actinomyces* spp.	1			1					
Other	1		1						
**Fungi**	11	(10)							
*Phaeohyphomycoses*	7					7			
*Candida species*	4		1	1	2				
**Parasites**	4	(4)							
*Cryptosporidium* spp.	3				3				
*Blastocystis hominis*	1				1				
**Not documented**	15	(14)	2	10	1		2		

* Note: ESBL-KP: Extended-Spectrum Beta-Lactamase producing *Klebsiella pneumoniae*; KP: *Klebsiella pneumoniae*; MSSA: Methicillin-Sensitive *Staphylococcus aureus*; UTI: Urinary Tract Infections; Wild type *E. coli*: non-resistant strain of *Escherichia coli*.

**Table 5:** Determinants of infection in the univariate and multivariate analysis.

	Case	Control	OR [95% CI]	*p* Value
Univariate analysis				
Female sex, No. (%)	25 (43.9)	5 (14.7)	3.97 [1.34–11.75]	0.005
Diabetes at time of transplant, No. (%)	17 (30.9)	2 (5.9)	6.37 [1.36–29.72]	0.007
Length of stay after transplantation, (days), median [IQR]	19.5 [14–30]	16 [12–24]	1.02 [20.9–71.07]	0.024
Multivariate analysis				
Female			4.33 [1.40; 13.37]	0.010
Diabetes mellitus at time of transplant			7.65 [1.59; 36.81]	0.011

Note. IQR: interquartile range; OR: odds Ratio.

## Data Availability

The original contributions presented in the study are included in the article, further inquiries can be directed to the corresponding author.
